# Going Home: Predictors of Reunification and Reentry in Dutch Child Protection Cases

**DOI:** 10.1177/10775595251409353

**Published:** 2026-01-16

**Authors:** Sabine van der Asdonk, Renate S. M. Buisman, Lenneke R. A. Alink, Anouk Goemans, Daisy J. H. Smeets, Jolien H. van Boven, Mariëlle R. Bruning

**Affiliations:** 1 Institute of Education and Child Studies, Leiden University, Leiden, The Netherlands; 2 Institute of Private Law, Leiden University, Leiden, The Netherlands; 3 Jeugdbescherming West, The Hague, The Netherlands

**Keywords:** reunification, out-of-home placement, child protection, reentry, risk factors

## Abstract

This study investigated predictors of reunification and reentry for children in Dutch out-of-home care. Case files of 340 children (aged 0–16) placed out of home in 2018 were coded and analyzed. Potential predictors were identified with bivariate correlations. A Cox proportional hazards model was used to predict the time until reunification, and a logistic regression to predict reentry. Within 6 years of placement, 42% of the children were reunified with their parents – of which 26% reentered out-of-home care. Professional support directed at the parents during placement more than doubled the likelihood of reunification. In contrast, multiple placement shifts, parental history of childhood maltreatment, limited parenting capacities, and parental intellectual disability decreased this likelihood. Among reunified children, parental childhood maltreatment, domestic violence, and placement in group care increased the risk of reentry in out-of-home care. Our findings underscore the importance of targeted professional support during and after the process of reunification.

## Introduction

Out-of-home placements are often initiated due to severe concerns about child abuse or neglect, but they can have profound effects on children’s development and have a big impact on the entire family system. In the Netherlands, as in many other countries, the child protection system faces the challenge of balancing child safety with the goal of family reunification (Child Welfare Information Gateway, 2011; Kooymans et al., 2023). Deciding whether and when to reunify a child with their family of origin is complex, as professionals have to balance the child’s rights and needs to further develop family life and grow up in a safe environment. Indeed, research has shown that a substantial number of reunified children are rereported to child protective services (Jones & Jonson-Reid, 2023) or reenter out-of-home care (e.g., Brown et al., 2020; Esposito et al., 2022). In addition, developmental outcomes for children who are reunified do not necessarily improve compared to children who remain in out-of-home care (Lindner & Hanlon, 2024). Understanding which factors influence the decision on reunification is thus crucial for improving the reunification process and subsequent outcomes for children and parents.

In the Netherlands, when a child’s development is severely at risk, the most far-reaching measure is to place the child out of home. While preferably, such placements occur on a voluntary basis, when this is insufficient or impossible, a court-mandated out-of-home placement can be imposed - often in combination with a family supervision order. The number of children with a court-mandated out-of-home placement has only been nationally monitored since 2022. This involved 10,045 children in that year (Centraal Bureau voor de Statistiek Statistics Netherlands., 2023). Although policy and legal frameworks emphasize the importance of reunification after placement, national numbers on reunification have been unavailable until now. A complicating factor is the decentralized Dutch youth care system, where child protection organizations each apply their own working methods and systems for recording case files.

Beyond the absence of national numbers on reunification, limited knowledge also exists on which children are most likely to be reunified with their parents, and which are at risk of reentering out-of-home care. Internationally, few studies outside of the United States have explored predictors of reunification (Sitjes-Figueras et al., 2025), and even fewer have examined predictors of subsequent reentry in out-of-home care. In the Netherlands, only one study has thus far thoroughly examined predictors of reunification - yet, the sample of this study was partly Flemish and only children placed in foster families were included (Goemans et al., 2016). This leaves an important knowledge gap, particularly given the considerable number of children placed in residential care in the Netherlands (Bruning et al., 2025). The current study is the first to provide nationally representative data on reunification and reentry among a broad sample of children placed in Dutch out-of-home care, and to identify predictors associated with these outcomes. Below follows a brief overview of factors that have been previously investigated in this context.

### Child Characteristics

Several child characteristics have been studied in relation to reunification, with mixed findings. Some studies suggest younger children are more likely to be reunified (e.g., Liming et al., 2021; López et al., 2013; Vanderfaeillie et al., 2017), while others report the opposite (e.g., Hélie et al., 2022; Lee et al., 2017; Lloyd et al., 2017), or no effect (Goemans et al., 2016; Van Holen et al., 2018). For gender, generally no effect is shown (e.g., Delfabbro et al., 2013; Goemans et al., 2016; Lee et al., 2017), with a few exceptions (e.g., Hélie et al., 2022). Findings on ethnicity are also inconsistent, though the majority of studies seem to report no effect (e.g., D’Andrade, 2017; Goemans et al., 2016; Hélie et al., 2022). For child behavior problems, a more complex picture arises from the literature. Studies generally show that the presence of behavior problems is not related to the likelihood of reunification (e.g., Goemans et al., 2016; López et al., 2013; Van Holen et al., 2018), though some studies report that behavior problems *during* placement decrease this likelihood (Goemans et al., 2016; Landers et al., 2019; Vanderfaeillie et al., 2017). Studies that looked at broader indices of child mental health also show mixed outcomes (e.g., Akin, 2011; Hélie et al., 2022; Van Holen et al., 2018). A final child factor with mixed findings concerns the presence of a disability or other health problem. Some studies find this decreases the likelihood of reunification (Akin, 2011; Lloyd et al., 2017), while others show no effect (López et al., 2013; Van Holen et al., 2018). In summary, while gender and ethnicity seem unrelated to reunification, child age, behavior problems, and health-related problems might play a role, warranting their inclusion as predictors in our study.

### Parent and Family Characteristics

Studies that have investigated parental alcohol or drug use as a predictor of reunification show mixed results. Several studies find a negative effect (e.g., Delfabbro et al., 2013; Hélie et al., 2022; Ryan et al., 2016), while other studies find no effect (Lloyd et al., 2017; Vanderfaeillie et al., 2017) or mixed effects (López et al., 2013). Similarly, psychological problems are sometimes negatively associated with reunification (Delfabbro et al., 2013; Hélie et al., 2022) and sometimes unrelated (Goemans et al., 2016; Van Holen et al., 2018). Parental incarceration generally does not lower the likelihood of reunification (e.g., Lloyd et al., 2017; Ryan et al., 2016; Van Holen et al., 2018), though one study found it to increase the likelihood of reunification for mothers (López et al., 2013). Research on parental intellectual disability and childhood maltreatment is more scarce, but thus far shows no effect (Hélie et al., 2022; Van Holen et al., 2018), though little is known about broader criminal history. Based on these findings, we included parental substance use, psychological problems, intellectual disability, childhood maltreatment, and criminal records as predictors in our study.

Demographic characteristics of the biological family have also been often investigated in relation to reunification. For poverty, unemployment, and housing problems, most studies report a negative effect (e.g., Delfabbro et al., 2013; Hélie et al., 2022; Lloyd et al., 2017), though some studies report the opposite (Lee et al., 2017; Van Holen et al., 2018). For single parenthood, similar variation exists (e.g., Lloyd et al., 2017; Van Holen et al., 2018). Given these results, we included financial problems, unemployment, housing problems, and parental separation as predictors in our study.

### Parenting Quality

The link between child maltreatment and reunification is complex. Physically abused children appear more likely to be reunified than neglected children, though the evidence is not entirely consistent (e.g., Akin, 2011; Hélie et al., 2022; Lee et al., 2017; Ryan et al., 2016). Although some studies find that interparental violence decreases the likelihood of reunification (Van Holen et al., 2018), others find no effect (Hélie et al., 2022). Comparable inconsistencies are reported for reduced parenting capacities (Delfabbro et al., 2013; Ryan et al., 2016; Van Holen et al., 2018). Therefore, we included parenting capacities and domestic violence (including child maltreatment) as predictors in our study.

### Placement Related Characteristics

A commonly investigated factor related to the out-of-home placement concerns the type of placement. A meta-analysis concluded that placement in kinship foster care does not affect reunification chances (Winokur et al., 2018), while excluded or later studies show mixed effects (e.g., Goemans et al., 2016; Landers et al., 2019; Ryan et al., 2016). Likewise, previous placements may reduce reunification chances (Akin, 2011; Lloyd et al., 2017; López et al., 2013) or have no effect (Goemans et al., 2016; Hélie et al., 2022; Van Holen et al., 2018). Placement with siblings generally seems to increase the likelihood of reunification (Akin, 2011; Hélie et al., 2022). For professional support during placement, one study reports no effect on reunification chances, regardless of whether the support was directed at the child, biological parent, or foster parent (Van Holen et al., 2018). Another study reports that when biological parents use professional support during placement, reunification chances increase (Goemans et al., 2016). However, other studies report contrary or mixed results (D’Andrade, 2017; Hélie et al., 2022; López et al., 2013). Finally, contact with biological parents during placement generally increases the likelihood of reunification (e.g., Goemans et al., 2016; Hélie et al., 2022; López et al., 2013). To conclude, though previous findings on contact with biological parents indicate that this increases the chance of reunification, findings on other placement related factors are mixed. In our study, we therefore included placement history (both prior out-of-home placements and number of placement shifts), placement with siblings, and professional support directed at biological parents as predictors. Even though we additionally aimed to include contact with the parents, this was not reported in a consistent and clear manner in the case files and was therefore excluded.

### Stability of Reunifications

Stability of reunification is often measured as the risk of reentry in out-of-home care after reunification. Existing research on reentry has been predominantly conducted in the United States (Davidson et al., 2019; Kimberlin et al., 2009), with few studies in other countries (i.e., in Canada (Esposito et al. (2022)) and the United Kingdom (Goldacre et al. (2022))). Given cross-national differences in child protection systems (Connolly & Katz, 2019), more research in different countries is needed, including in the Netherlands. Below follows a brief summary of relevant predictors that have emerged from previous studies.

#### Child Characteristics

Older children are generally less likely to reenter out-of-home care following reunification (e.g., Brown et al., 2020; Font et al., 2018; Wulczyn et al., 2020). Although most studies report that girls have lower odds to reenter out-of-home care (e.g., Ahn et al., 2025; LaBrenz et al., 2020; Wulczyn et al., 2020), other studies report no gender effects (e.g., Font et al., 2018). Even though behavioral and academic difficulties have been associated with higher reentry rates, the evidence is limited (Ahn et al., 2025; Esposito et al., 2022; Font et al., 2018). With respect to ethnicity, most studies show that white children have lower reentry rates (e.g., Ahn et al., 2025; Brown et al., 2020; Wulczyn et al., 2020). Because ethnicity could not be reliably coded in our study, we did not include this variable. We did include child age, gender, behavioral problems, and health problems as predictors of reentry.

#### Parent and Family Characteristics

Single parenthood (Shaw, 2006) and poverty (Brown et al., 2020) have been found to increase the risk of reentry, though the evidence is sparse. Substance use has been more consistently associated with the risk of reentry (Esposito et al., 2022; LaBrenz et al., 2020; Shaw, 2006). Mixed results are reported for type of maltreatment and parenting capacities (LaBrenz et al., 2020; Shaw, 2006; Wells & Guo, 1999). These five factors were therefore included as predictors of reentry in this study.

#### Placement Related Characteristics

Children who have experienced previous removals (Ahn et al., 2025; Jedwab & Shaw, 2017; LaBrenz et al., 2020) and multiple placement shifts (Brown et al., 2020; Courtney, 1995; Wells & Guo, 1999) are more likely to reenter out-of-home care. With respect to placement setting, research overwhelmingly shows that children placed in kinship foster care are less likely to reenter out-of-home care (Brown et al., 2020; Courtney, 1995; Font et al., 2018; Koh & Testa, 2011; Shaw, 2006). One study reports that this effect can be partly explained by a lower amount of placement shifts for these children (Koh & Testa, 2011). One study additionally finds that children in group care are even more likely to reenter out-of-home care than children in non-kinship foster families (Wells & Guo, 1999).

#### Time In Out-Of-Home Care

Most studies report that a longer time in out-of-home care before reunification results in lower reentry rates (e.g., Brown et al., 2020; Wulczyn et al., 2020). However, one study finds the opposite (Font et al., 2018) and in one study this only applies to very short (3 months or less) placement periods (Courtney, 1995). Another study does not report any effects of time in out-of-home care (Ahn et al., 2025). Therefore, this factor was included as a predictor of reentry in our study.

### Current Study

The goal of this study was to investigate (1) which factors predict reunification of children after out-of-home placement and (2) which factors predict reentry in out-of-home care for these children. We analyzed case files of children in the Netherlands placed in out-of-home care under a court-mandated child protection order. This is the first report that investigates reunification trajectories in a broad sample of children in Dutch out-of-home care.

## Methods

### Sample

In total, 340 case files from seven child protection organizations throughout the Netherlands (the total number of Dutch child protection organizations is 14) were analyzed. This study was part of a larger research project concerning the process of out-of-home placement and reunification in Dutch child protection cases (Bruning et al., 2025).

#### Selection of Case Files

Inclusion criteria for case files in the overarching study were: (1) the case file involved a mandatory out-of-home placement with a child protection order, (2) the child was 0–18 years old, (3) the placement was mandated in 2018, and (4) the mandated out-of-home placement was actually carried out. In some of the participating organizations, case files were structured at the family level and could include multiple children from the same family. In these cases, one child was randomly selected. We used stratified randomized sampling to ensure a representative selection of case files. Each organization provided a list of eligible cases, and the number of selected files was proportional to the organization’s total caseload. Microsoft Excel was used for random selection.

The original sample from the larger research project involved 456 case files. For the purpose of the current report, additional exclusion criteria were applied. First, secure treatment placements were excluded, because of fundamental differences in legal status and intervention approach with other types of placement. Second, cases that involved a placement with the other parent were excluded, because this is a rather unique and uncommon type of placement. Third, children aged 17 years were excluded since they nearly aged out of care and support for this group focuses more on developing independence than on reunification. Finally, children were excluded if they “returned” to their other parent (i.e., not the parent they lived with prior to placement). This resulted in a final sample of 340 case files involving children aged 0–16 years who were initially placed in a residential facility (25%), family-style group care (6%), or a kinship or non-kinship foster family (31% and 38%, respectively).

#### Procedure

This study was approved by the research ethics committee of the research ethics committee of the Institute of Education and Child Studies from Leiden University (case number ECPW-2022/374). We emailed ten child protection organizations with an information letter. Seven organizations agreed to participate. Coding took place between July 2023 and March 2024 by Master’s students in Education and Child Studies, who were supervised by one of the researchers. The case files concerned children who were placed out-of-home in the year 2018. Reunifications and subsequent reentries were monitored until the date of coding, or until the child turned 18 (i.e., the age at which child protection measures terminate by law). This covered a time span between 24 and 74 months (*M* = 56.57, *SD* = 14.70).

#### Coding System

Due to the decentralized nature of the Dutch child protection system, case records are not maintained in a standardized manner. Each child protection organization employs its own system for keeping these records – for example, some organize files around individual children, whereas others do so around families. Moreover, no overarching system exists that allows for systematic retrieval of the data relevant to this study. Therefore, coding had to be conducted manually. We first developed a coding system which we tested on a pilot sample of case files from one organization. We then made adjustments to improve feasibility and ensure reliability (e.g., simplifying or removing variables that were challenging to code objectively). We used two standard documents that were present in all case files for coding: the most recent ‘family plan’ written by the child protection organization and the investigation report from the Child Care and Protection Board supporting the placement order request to the court. Risk factors were coded as present only when documented by a professional (i.e., not solely based on parental statements) and when applicable *at the start of* placement. Intellectual disability was only coded if an official diagnosis was reported. Limited parenting capacities were coded as present if this was mentioned in the court-mandated child protection order. If no information was available on a certain risk factor, it was conservatively recoded as 0 (absence). This approach was chosen because case records usually only described risk factors when they were present in that particular case. As a result, risk factors were rarely coded as absent in practice. Parental risk factors were initially coded separately for mothers and fathers, but were combined into one dichotomous score, where 1 indicated the presence of the risk factor in at least one parent and 0 indicated its absence in both.

Of the variables included in this report (see Table 1), the following child characteristics were coded: age (in years), behavioral problems, health problems (e.g., diabetes or blindness), and intellectual disability. Parent characteristics included psychological problems (e.g., depressive episodes, anxiety), intellectual disability, substance use, history of criminal behavior (evidenced by past convictions or suspected crimes), limited parenting capacities, and experienced childhood maltreatment. At the family level, we coded whether the biological parents were separated and whether financial problems, unemployment, housing problems, and domestic violence (encompassing any type of maltreatment or other domestic violence) were present. Finally, the following placement-related characteristics were coded: prior out-of-home placement, number of placement shifts since the start of the placement (e.g., shifts between foster families or from group care to foster care), and initial placement without siblings (coded as 0 = placement with siblings or not applicable if no siblings were present or placed out of home, 1 = placement without siblings). We also included initial placement setting, operationalized in two separate variables: (1) placement in a kinship or non-kinship foster family or family-style group care [in Dutch: “gezinshuis”] relative to placement in (other types of) residential care, and (2) initial placement in a kinship foster family, relative to all other settings. Professional support for the biological parents was coded as present if an intervention was provided that targeted the family system or parent-child interaction (such as Families First or a video-feedback intervention) and/or the parent (such as trauma therapy).

**Table 1.** Descriptive Statistics of the Sample

	Complete sample (*N* = 340)	Reunified versus non-reunified children		Reunified children with and without reentry	
		Reunified (*n =* 144; 42%)	Non-reunified (*n* = 196; 58%)	No reentry (*n* = 106; 74%)	Reentry (*n* = 38; 26%)
	%/*M(SD)*	%/*M(SD)*	%/*M(SD)*	%/*M(SD)*	%/*M(SD)*
Child characteristics					
Age (in years)	8.17 (5.59)	8.19 (5.21)	8.15 (1.30)	7.84 (5.05)	9.18 (5.61)
Gender (% boys)	53%	51%	54%	49%	58%†
Behavioral problems	47%	51%	44%	47%†	63%†
Health problems	7%	9%	6%	7%†	16%†
Intellectual disability	15%	16%	14%	13%	24%
Parent and family characteristics					
Psychological problems	58%	57%	59%	54%	66%
Intellectual disability	25%	19%†	29%†	19%	21%
Substance use	37%	33%	40%	33%	32%
Criminal behavior	20%	20%	20%	20%	21%
Childhood maltreatment	19%	14%*	24%*	10%*	24%*
Limited parenting capacities	87%	83%†	89%†	82%	84%
Biological parents separated	78%	79%	78%	79%	76%
Financial problems	46%	44%	47%	40%†	55%†
Housing problems	26%	25%	27%	26%	24%
Domestic violence	58%	58%	58%	52%*	74%*
Placement characteristics					
Prior placement	35%	29%*	40%*	31%	24%
N placement shifts	1.01 (1.48)	0.62 (1.12) **	1.30 (1.65)**	0.44 (0.79)*	1.11 (1.64)*
Placement in family setting^a^	75%	72%	78%	80%**	47%**
Placement in kinship family^b^	31%	33%	30%	38%*	18%*
Placement with siblings (or n.a.)	77%	76%	79%	80%*	63%*
Support aimed at parents	66%	79%**	56%**	82%	71%
Months in out-of-home care				13.09 (13.15)	9.61 (9.33)

*Note.* Chi-square tests and independent t-tests were used to test for differences between groups; † <.10; * <.05; ** <.01.

^a^Coded as 1 = foster family (kinship or non-kinship) or family-style group care, 0 = all other types of residential care.

^b^Coded as 1 = kinship foster family, 0 = all other types of placements.

Reunification was defined as the child returning to at least one parent with whom the child resided prior to out-of-home placement. This definition applied regardless of whether the reunification was planned or unplanned, and regardless of when it occurred, as long as it took place before the child turned 18 and the child protection measures were legally terminated. Reentry was defined as any out-of-home placement occurring after reunification.

Two groups of in total 14 Master’s students in Education and Child Studies were trained to use the coding system. Training involved a thorough review of the coding manual and collaborative coding of practice files. The students then independently coded a reliability set. Due to practical constraints, each group coded a separate set of 15 case files, and one research assistant ensured consistency. Because most variables were dichotomous and the sets were small, we assessed reliability using percentage agreement instead of Cohen’s kappa to avoid bias (McHugh, 2012). An average inter-rater agreement of 80% per variable was considered sufficient (McHugh, 2012); see Supplemental material for more details. For child age, intraclass correlation coefficients (single measures, absolute agreement) between coder pairs ranged from .94-1.00, indicating excellent reliability (Koo & Li, 2016).

For four variables, the average inter-rater agreement fell below 80%. To assess potential discrepancies in data quality for these variables, we conducted Chi-square tests and t-tests to compare data coded by less reliable coders to data coded by reliable coders. No significant differences were found for prior placements, number of placement shifts, and professional support aimed at biological parents, confirming consistency across coders on these variables. Only for unemployment, systematic differences across coders were found (χ^2^ (1) = 6.46, *p* = .011). Therefore, this variable was eliminated from further analyses.

### Statistical Analyses

All variables included in the analyses had complete data. For the number of placement shifts, three outliers were winsorized to the next highest value within an acceptable range (*z* < 3.29). Given the sample size, the study lacked sufficient power to include all variables in multivariate analyses. Therefore, we applied a data-driven approach, selecting potentially relevant predictors based on bivariate Spearman’s rho correlations (*r* > .10, *p* < .10). To investigate which factors predicted reunification, we conducted a multilevel Cox proportional hazards model that accounted for the nesting of children within organizations, using the *survival* and *coxme* packages in R Version 4.4.1 (Therneau, 2024a, 2024b). The event time was defined as the time until reunification or until the date of file coding (in months). Significant predictors were visualized using Kaplan-Meier curves, using the *ggplot2*, *survival*, and *survminer* packages (Kassambara et al., 2024; Therneau, 2024a; Wickham, 2016). To explore which factors predicted reentry in out-of-home care, a similar approach was used to identify relevant predictors. Due to the absence of data on duration until reentry, we conducted a multilevel logistic regression model for this binary outcome variable (reentry versus no reentry) using the *lavaan* and *lme4* packages (Bates et al., 2015; Rosseel, 2012).

## Results

### Descriptive Statistics

Table 1 presents descriptive statistics of the sample. Forty-two percent of the children were reunified within 6 years of placement. Reunification occurred on average 12.05 months after placement (*SD* = 12.57), with a range between <1 month and 60 months. Variables that correlated with reunification at *r* > .10 and *p* < .05 were included as predictors in the multivariate analyses (see Supplemental material). Reunified children more often had parents with no history of childhood maltreatment, had no prior placement, had fewer placement shifts, and had parents who received professional support (*p* < .05). In addition, their parents less often had limited parenting capacities or an intellectual disability (*p* < .10).

Of the reunified children (*n* = 144), 26% reentered out-of-home care within the study period. Similar as for the predictors of reunification, variables with a correlation at *r* < .10 and *p* < .05 were included as predictors of reentry (see Supplemental material). Children who reentered out-of-home care more often had parents with a history of childhood maltreatment, had been exposed to domestic violence, had experienced more placement shifts, were more often placed in residential or non-kinship settings, and were less often placed with their siblings (*p* < .05). They also more often showed behavioral and health problems (*p* < .10).

### Predictors of Reunification

**Figure 1.**
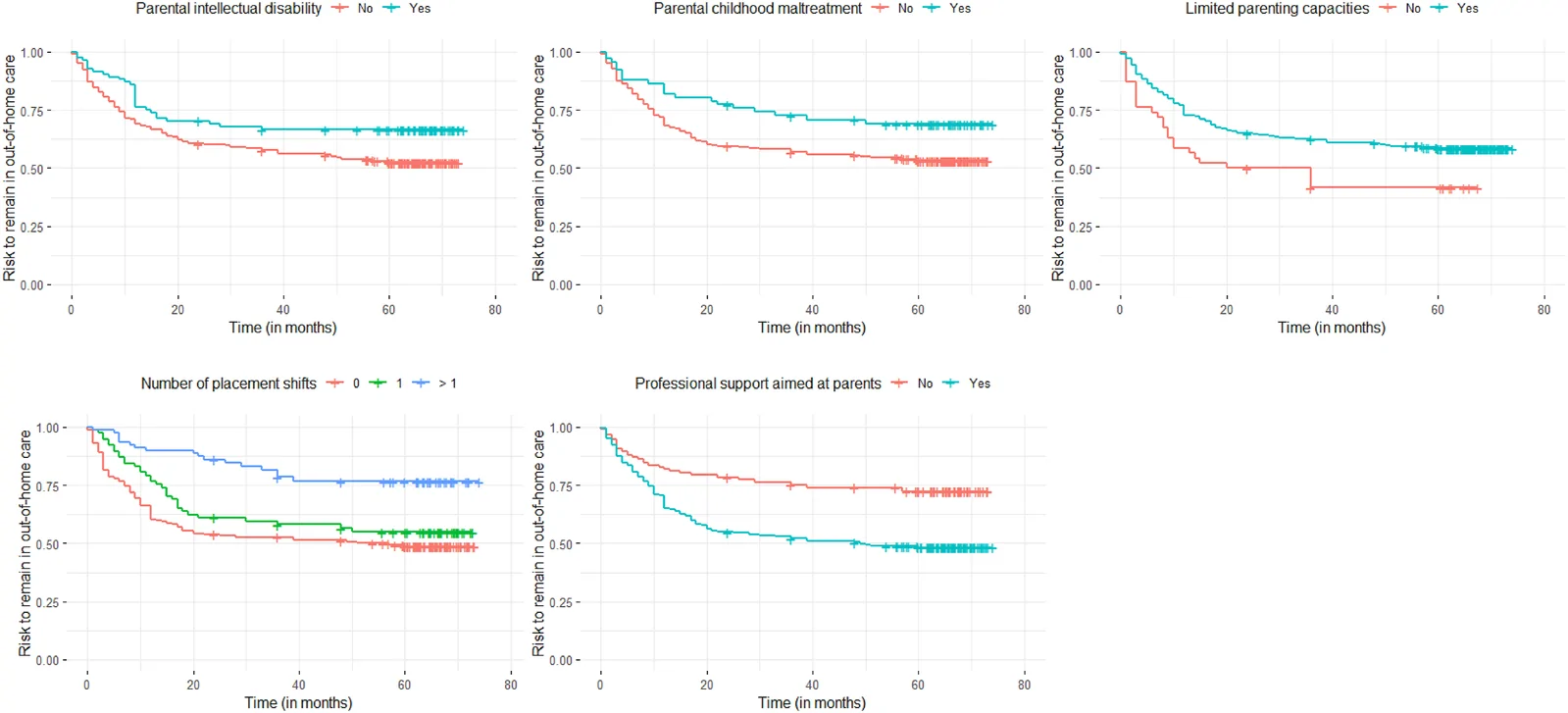
Kaplan-Meier survival plots displaying the univariate associations between significant predictors and the likelihood of reunification (presented in opposite direction on the *Y*-axis as the risk of remaining in out-of-home care) (*N* = 340)

The results of the Cox proportional hazards model predicting time until reunification are shown in Table 2. The intraclass correlation coefficient (ICC) was .02, indicating that only 2% of the variance was attributable to differences between child protection organizations. The findings reveal that, when controlling for other included predictors, the strongest predictor of reunification was professional support aimed at the parents (*Hazard Ratio [HR]* = 2.17, *p* < .01). When such support was provided during placement, the likelihood of reunification more than doubled. Moreover, each placement shift experienced by the child decreased reunification chances by 28% (*HR* = 0.72, *p* < .01). Parental history of childhood maltreatment reduced the likelihood of reunification by 45% (*HR* = 0.55, *p* = .02), and the presence of limited parenting capacities reduced this likelihood by 42% (*HR* = 0.58, *p* = .02). Children whose parents had an intellectual disability had 38% lower chances of reunification (*HR* = 0.62, *p* = .03). Although prior placement of the child was associated with reunification in the bivariate analyses, it was not a significant predictor in the multivariate model. Figure 1 illustrates that group differences remained consistent over time. For parental history of childhood maltreatment and professional support, the effects appeared only after a few months and became more pronounced over time. To visualize the effect of placement shifts, we created a categorical variable reflecting the number of placement shifts, which shows that especially children who experienced more than one shift had substantially lower reunification chances.

**Table 2.** Cox Proportional Hazards Model Estimating the Time Until Reunification (*N *= 340)

	*HR*	95% CI
Parental intellectual disability	0.62*	−0.91; −0.05
Parental childhood maltreatment	0.55*	−1.09; −0.11
Limited parenting capacities	0.58*	−0.99; −0.09
Prior placement	0.74	−0.67; 0.07
*N* placement shifts	0.72**	−0.50; −0.17
Professional support aimed at parents	2.17**	0.36; 1.18

χ2 (7.97) = 64.73, *p* < .01.

*p < .05, **p < .01.

### Predictors of Reentry

A multilevel logistic regression model predicting reentry in out-of-home care could not be estimated due to convergence issues, caused by insufficient variance at the child protection organization level. We therefore employed a logistic regression model without multilevel structure (see Table 3). Three out of the eight included predictors remained significant after controlling for the effects of other variables. The risk of reentry was higher when parents had a history of childhood maltreatment (*Odds Ratio [OR]* = 4.06, *p =* .02), when domestic violence had taken place (*OR* = 3.39, *p* = .01), and lower when the child had been placed in a family setting (*OR* = 0.12, *p* < .01). The largest effect size was found for placement setting, though the small sample size precludes drawing strong conclusions from this finding.

**Table 3.** Logistic Regression Model With Reentry as Outcome Variable (*N *= 144)

	*B*	*SE*	*OR*	95% CI
Intercept	−0.51	0.72		
Child behavioral problems	−0.50	0.55	0.61	0.20; 1.75
Child health problems	0.95	0.75	2.58	0.58; 11.60
Parental childhood maltreatment	1.40*	0.61	4.06	1.22; 13.70
*N* placement shifts	0.38^†^	0.22	1.46	1.00; 2.26
Domestic violence	1.22*	0.49	3.39	1.34; 9.40
Placement in family setting	−2.10**	0.64	0.12	0.03; 0.41
Placement in kinship foster care	0.08	0.60	1.08	0.33; 3.50
Placement with siblings	−0.42	0.47	0.66	0.26; 1.67

†*p* < .10, *p < .05, **p < .01.

## Discussion

This study is the first report that investigated reunification trajectories of children in Dutch out-of-home care. Over a period of approximately 6 years, 42% of the children were reunified with their parents – of which 26% later reentered out-of-home care. Children were more likely to be reunified if professional support was provided to the parent during placement. Conversely, the likelihood of reunification decreased for each additional placement shift the child had experienced, and for children whose parents had a history of childhood maltreatment, limited parenting capacities, or an intellectual disability. When exploring the factors predicting reentry in out-of-home care, we found that children were more likely to reenter if their parents had a history of childhood maltreatment, if domestic violence had occurred in the family, or if the child had been placed in a group care setting.

In previous studies, a variety of reunification percentages ranging from 8 to 86% have been reported (Sitjes-Figueras et al., 2025). However, these studies cover a wide range of time periods and used varying operationalizations of the construct reunification (e.g., some also including other types of permanency such as adoption), making it hard to compare these percentages. The previous study that included a partly Dutch sample of children in foster families reported a percentage of 15.9% for the Dutch subsample (Goemans et al., 2016). This study analyzed the case files over a roughly similar time period as the current study, though it was conducted about a decade earlier and included both mandatory and voluntary placements in foster families. Given the recent developments in the Dutch child protection context, with a more pronounced focus on reunifying children (as illustrated by recently updated guidelines for professionals which explicitly state reunification is a key goal, see Kooymans et al., 2023), it is not surprising that the percentage reunified children in our sample is higher. Fewer prior studies have examined reentry rates, but the studies that have been conducted show a range between 14–54% (e.g., Australian Institute of Health and Welfare, 2024; Brown et al., 2020; Esposito et al., 2022; Goldacre et al., 2022; Wulczyn et al., 2020). Our reentry rate of 26% falls within this range; however, here too differences in study periods and other methodological factors hinder making direct comparisons. Either way, efforts should be made to reduce this number, considering the negative impact of moving in and out of out-of-home care and possibly reexperiencing child maltreatment in the home environment again (Jones & Jonson-Reid, 2023). Strikingly, 35% of the children in our sample already had experienced a (mandatory or voluntary) out-of-home placement prior to this study. A hopeful note is that visitation by a social worker post-reunification can decrease the odds of reentry in out-of-home care (Ahn et al., 2025; Jedwab & Shaw, 2017).

The strongest predictor of reunification in our study was professional support provided to the parents (either focused on their parenting or individual problems) during placement: The likelihood of reunification more than doubled for families that had received such support. Although this result is in line with a previous study that involved a partly Dutch sample (Goemans et al., 2016), other studies did not find this effect (e.g., D’Andrade, 2017; Hélie et al., 2022; López et al., 2013). This finding could imply that supporting parents in their parenting or in coping with their personal problems actually increases the chance of reunification. At the same time, it could also indicate a selection effect, where families who already have a higher likelihood of reunification (for instance because the parents have a stronger working alliance with their case worker) more likely receive such support. The varying quality of case files and differences between organizations hindered more detailed coding of the type of interventions that were provided (e.g., attachment-based interventions or interventions focused on more general parenting skills). Future research, preferably using longitudinal, experimental designs, is needed to better understand what works for whom. The limited available evidence suggests that family-focused interventions targeting the parent-child relationship are more effective than individual interventions (Maltais et al., 2019).

A second predictor that emerged from our study concerns placement shifts: children who experienced placement shifts were less likely to be reunified. This effect was most pronounced for children who experienced multiple placement shifts. Most previous studies did not find such an effect, though these mostly focused on children placed in foster families (Goemans et al., 2016; Hélie et al., 2022; Van Holen et al., 2018; Vanderfaeillie et al., 2017) – while placement in a group care setting is associated with high placement instability rates (Leloux-Opmeer et al., 2016; Riemersma et al., 2023). Children who experience placement instability are more likely to show emotional and behavioral problems and other negative developmental outcomes. This association likely works in two directions. On the one hand, the presence of more severe psychopathology increases the risk of placement instability. On the other hand, placement shifts – especially when children are not involved in or even timely informed about the decision (Hyde & Kammerer, 2009) – can themselves be traumatic events that exacerbate behavioral problems (Asif et al., 2024; Maguire et al., 2024; Newton et al., 2000). Even though no causal conclusions can be derived from our finding, efforts should clearly be made to reduce the number of placement shifts, for instance by improving the matching process and providing adequate support to the child’s caregivers during placement (e.g., Konijn et al., 2021).

Parental traumatic childhood experiences and intellectual disability additionally decreased the likelihood of reunification in our study. Even though the few studies that included these factors do not support these findings (Hélie et al., 2022; Van Holen et al., 2018), there is substantial evidence that parents who have experienced maltreatment in their childhood are more likely to maltreat their own children (Madigan et al., 2019). Prior studies have identified mothers with traumatic childhood experiences as a particularly vulnerable group that is less likely to benefit from parenting interventions (Moran et al., 2005; Steele et al., 2019; Van der Asdonk et al., 2021). Even though our finding needs to be replicated, sufficient attention should be paid to experiences of childhood trauma in parents involved in child protection services – and adequate therapy and support should be provided to reduce the potential impact on parenting (Van Ee & Meuleman, 2024).

Previous studies have shown that children from parents with an intellectual disability are more often placed out-of-home, which can only be partly explained by shared risk factors such as poverty or parental mental problems (McConnell et al., 2021). Parental custody is also terminated more often for these families (Tøssebro et al., 2017). In our study, parental intellectual disability decreased the likelihood of reunification, independent of the effect we found for limited parenting capacities. This seems to be in line with the observation that parents with an intellectual disability are – after controlling for other risk factors – less often referred for matched professional support services and even four times less likely to be referred to services aimed at working towards reunification (Pacheco et al., 2022). As illustrated by a study examining social workers’ perspectives, the assessment of “good enough parenting” in parents with an intellectual disability is a highly complex task for which more research would be desirable (Norlin & Randell, 2023).

Our analyses concerning the factors that predict reentry in out-of-home care, which were more explorative due to the smaller sample size, revealed three significant factors. Parental history of childhood maltreatment was associated with both reunification likelihood and reentry rates. This further underscores the importance of addressing traumatic childhood experiences in parents. Furthermore, the presence of domestic violence at the start of placement predicted a higher risk of reentry. This contrasts with an earlier study (Esposito et al., 2022) and thus requires further research. However, the finding is in line with the often chronic nature of domestic violence in families (e.g., Doelman et al., 2025) and stresses that these families should be carefully monitored and supported post-reunification.

Our finding that children placed in group care more often reentered out-of-home care is in line with some earlier studies (Wells & Guo, 1999) – though one only found this effect for children who did not experience prior removals (Jedwab & Shaw, 2017). Many other studies have shown that children in kinship foster care are less likely to reenter out-of-home care (e.g., Brown et al., 2020; Font et al., 2018; Koh & Testa, 2011). Although we found a significant bivariate correlation, we did not replicate this finding in our multivariate analyses. Similarly, the fact that we did not find that children’s age or the time they had been in out-of-home care predicted their risk of reentry, is not in line with the majority of earlier reports. That is, these predominantly showed that children are less likely to experience a stable reunification if they have been in out-of-home care for a shorter time period (e.g., Brown et al., 2020; Shaw, 2006; Wulczyn et al., 2020) and if they are older (e.g., Ahn et al., 2025; Font et al., 2018; Wulczyn et al., 2020). All of these earlier studies were conducted outside of Europe – further underscoring the importance of conducting studies in different countries and continents.

### Implications

This study has several implications for child protection research and practice. In line with the basic principle that every child has the right to family life, child protection services should maximize efforts to increase the rate of stable reunifications. Even though our findings do not permit causal conclusions, professional support during placement - targeting both parenting and parents’ personal difficulties - seems to be a key element. Parents with histories of childhood trauma appear especially vulnerable, as their children were less likely to be reunified and more likely to reenter care. Professionals involved with families in child protection should be aware of the potential impact of parental trauma histories and when indicated, offer trauma-informed interventions, which preferably integrate parenting support with trauma treatment (Van Ee & Meuleman, 2024).

Equally important is the period after reunification. Child protection services should prioritize providing more support and careful monitoring of families to reduce reentry rates. At the same time, longitudinal studies are needed to clarify which intervention strategies work best for whom. Future research should also examine the role of parent-child contact during placement, and use more nuanced assessments of risk and protective factors (e.g., validated questionnaires or interviews to assess parental psychopathology). Finally, in the Dutch context, despite the availability of national guidelines, practices and record-keeping vary greatly across organizations due to the decentralized child protection system. More standardization could improve transparency and facilitate routine evaluation of reunification and reentry trends, which can ultimately contribute to improved policy and practice.

### Limitations

Some limitations of this study should be noted. Data collection was dependent on the case files from different child protection organizations, each with its own structure. Because there is no standardized system across these organizations, developing a coding system that could be reliably used to code all relevant data was complex. On the other hand, the low ICC in our analyses (.02) shows that differences between child protection organizations barely contributed to differences in the time until reunification. A second limitation is that the labor-intensive nature of the coding process constrained the sample size – because of which particularly the analyses concerning the stability of reunification should be interpreted with caution. Also, it is possible that relevant information was overlooked if it was not documented by the involved professionals or if the coders could not find this information. In such cases, we classified the risk factor as absent. While this conservative approach reduced the chances of false positives, it also implies that the prevalence of risk factors in our sample is likely underestimated. We aimed to increase the validity as much as possible by conducting a pilot and consulting professionals from the child protection organizations to ensure clarity on coding procedures (e.g., where to find certain types of information or which terminology was used by the organization) – which at least resulted in good interrater reliability.

It is also worth noting that the study sample consists of children placed in 2018, prior to significant policy changes in the Netherlands – such as updated guidelines for out-of-home placements with a stronger emphasis on reunification (Kooymans et al., 2023). Current practices may thus differ from those reflected in this retrospective sample. Another contextual factor concerns the potential impact of COVID-19. The children in our sample entered out-of-home care in 2018, and in the spring of 2020 the Netherlands went in lockdown. During this period, child protection organizations reported suspensions of supervised parent-child contact (Nederlands Jeugdinstituut [Netherlands Youth Institute], 2021). Admissions to, and transitions within, residential care facilities were restricted, and daily activities for youth residing there often stopped – which challenged group dynamics. These disruptions may have (negatively) affected reunification and reentry rates in our sample. However, because a large part of reunifications occurred within the first 12 months, before the lockdown, the impact on our findings – at least with respect to reunifications – is likely limited.

### Conclusion

To conclude, we found that 42% of the children were reunified with their parents within 6 years after placement, but one in four of these children later reentered out-of-home care. Reunification was twice as likely to occur when parents received professional support during placement, and less likely when children experienced multiple placement changes or when parents had a history of childhood maltreatment, an intellectual disability, or limited parenting capacities. Reentry risk was higher for children exposed to domestic violence, placed in a residential setting, or with parents who had experienced childhood maltreatment. These findings can inform child protection policies, for instance by incorporating predictors of reunification and reentry in guidelines for child protection workers. While more research is needed to identify effective intervention approaches, our results emphasize the importance of tailoring interventions to families’ individual needs during placement and after reunification. This way, ultimately more children can grow up safely, ideally within their own family.

## Supplemental Material

Supplemental Material - Going Home: Predictors of Reunification and Reentry in Dutch Child Protection Cases Supplemental Material for Going Home: Predictors of Reunification and Reentry in Dutch Child Protection Cases by Sabine van der Asdonk, Renate S. M. Buisman, Lenneke R. A. Alink, Anouk Goemans, Daisy J. H. Smeets, Jolien H. van Boven, and Mariëlle R. Bruning in Child Maltreatment

## Data Availability

Data will be made available upon reasonable request.*
