# Supplementary material for: Going Home: Predictors of Reunification and Reentry in Dutch Child Protection Cases
Source: Child Maltreat. 2026 Jan 16;31(4):739–50. doi: 10.1177/10775595251409353 (PMC13616033; doi:10.1177/10775595251409353)
Supplement: Supplementary file 1 [file sj-pdf-1-cmx-10.1177_10775595251409353.pdf]

Supplementary material

Supplementary Table 1

Means and Ranges of Percentages Agreement Between all Coder Pairs

	M	*Min*	*Max*
Child characteristics			
Behavioral problems	80%	60%	93%
Chronic disease or physical handicap	95%	87%	100%
Intellectual disability	94%	87%	100%
Parent characteristics			
Psychological problems	84%	67%	100%
Intellectual disability	96%	87%	100%
Substance use	88%	60%	100%
History of criminal behavior	90%	67%	100%
Experiences of childhood maltreatment	82%	60%	100%
Limited parenting capacities	80%	60%	100%
Family characteristics			
Biological parents divorced or separated	100%	100%	100%
Financial problems	80%	53%	100%
Unemployment	77%	60%	
Housing problems	88%	60%	100%
Domestic violence	81%	60%	100%
Removal related characteristics			
Prior placements	76%	60%	100%
N placement shifts	77%	60%	100%
Placement in family setting	86%	73%	100%

Placement in kinship foster care	88%	73%	100%
Placement with siblings	81%	60%	100%
Professional support aimed at biological parent(s)	72%	47%	93%

Supplementary Table 2

Bivariate Spearman Correlations Between All Study Variables (N = 340)

	1	2	3	4	5	6	7	8	9	10	11	12	13	14	15	16	17	18	19	20	21
1.Reunification	-																				
2.Child behavioral problems	.07	-																			
3.Child intellectual disability	.03	.26**	-																		
4.Child age	-.00	.49**	.32**	-																	
5.Child health problems	.07	.04	.11*	-.07	-																
6.Parental psychological problems	-.02	-.12*	-.22**	-.23**	.07	-															
7.Parental intellectual disability	-.11†	-.12*	-.03	-.21**	.00	.07	-														
8.Parental substance use	-.07	-.13*	-.20**	-.24**	-.02	.30**	.09	-													
9.Parental criminal behavior	-.00	-.11	-.07	-.24**	.00	.13	-.00	.21	-												
10.Parental childhood maltreatment	-.12*	-.03	.03	-.15**	.07	.16**	.10†	.24**	.12*	-											

11.Limited parenting capacities	-.10†	-.13*	-.01	-.29**	.11*	.19**	.13*	.14**	.01	.19**	-										
12.Biological parents separated	.01	.07	-.00	.07	-.05	.07	-.01	-.03	.04	-.03	-.06	-									
13.Financial problems	-.04	-.10†	-.08	-.15**	-.02	.15**	.08	.13*	.18**	.15**	.14**	-.00	-								
14.Housing problems	-.02	-.13*	-.11*	-.28**	-.11*	.10†	.07	.08	.17**	.10†	.08	.02	.24**	-							
15.Domestic violence	.00	.05	-.03	-.01	-.02	.17**	.04	.15**	.11*	.08	.17**	-.05	.04	-.01	-						
16.Prior placement	-.11*	.13*	.15**	.21**	-.01	-.07	-.08	-.04	-.01	.06	-.09	.14*	-.03	-.07	.01	-					
17.*N* placement shifts	-.22**	.26**	.04	.23**	.09	-.06	-.10†	-.00	-.14*	-.08	.03	.06	-.09	-.03	.03	.02	-				
18.Placement in family setting	.08	-.38**	-.30**	-.52**	-.06	.18**	.17**	.18**	.15**	.09†	.27**	-.06	.14**	.21**	.02	-.11	-.24**	-			
19.Placement in kinship family	.03	-.01	-.08	-.00	-.09	.04	-.03	.11*	.14*	.03	.10†	.09†	.04	.13*	.08	.08	-.25**	-.38**	-		
20.Placement with siblings (or n.a.)	-.03	-.15**	-.11*	-.23**	-.18**	.14*	.10†	.11*	.06	-.02	.01	-.01	-.01	.13*	-.02	-.01	-.02	.11*	-.03	-	
21.Support aimed at parents	.24**	-.09†	-.02	-.17**	.01	.22**	.13*	.10†	.09	.10†	.08	-.08	.07	.07	.06	-.07	-.13*	-.12*	-.04	.13†	-

† $p < .10$, * $p < .05$, ** $p < .01$

Supplementary Table 3

Bivariate Spearman Correlations Between All Study Variables and Reentry (N = 144)

	r_s
Child gender	-.08
Child behavioral problems	.14†
Child intellectual disability	.13
Child age	.10
Child health problems	.14†
Parental psychological problems	.11
Parental intellectual disability	.02
Parental substance use	-.01
Parental criminal behavior	.01
Parental childhood maltreatment	.17*
Limited parenting capacities	.03
Biological parents separated	-.03
Financial problems	.14
Housing problems	-.02
Domestic violence	.19*
Prior placement	-.07
N placement shifts	.18*
Placement in family setting	-.32**
Placement in kinship family	-.18*
Placement with siblings (or n.a.)	-.18*
Support aimed at parents	-.12
Time until reunification	-.12

† p <.10, * p <.05, ** p <.01
